# Reinfection or relapse? A case study of whole genome sequencing guided genomic characterization of *Mycobacterium abscessus* chronic infection in a cystic fibrosis patient

**DOI:** 10.1016/j.idcr.2022.e01491

**Published:** 2022-03-28

**Authors:** Rachit Chawla, Benjamin von Bredow, Jaime Deville, Shangxin Yang

**Affiliations:** a University of California, Los Angeles, CA, USA; b Harbor-UCLA Medical Center, Los Angeles, CA, USA; c Department of Pathology and Laboratory Medicine, UCLA David Geffen School of Medicine, Los Angeles, CA, USA; d UCLA Mattel Children's Hospital, Los Angeles, CA, USA

**Keywords:** Whole genome sequencing, *Mycobacterium abscessus*, Genomic characterization, Relapse, Reinfection, Cystic fibrosis, Drug resistance, Phylogenetic analysis

## Abstract

A 7-year-old cystic fibrosis patient with increased cough, new pulmonary infiltrate, and declining pulmonary function was diagnosed with clarithromycin resistant *Mycobacterium abscessus* infection. Treatment was initiated with clofazimine, linezolid and cefoxitin; she responded well to therapy and achieved microbiological clearance after completion of 12-month treatment. One year later, she had re-emergence of worsening symptoms and her sputum culture again grew clarithromycin resistant *M. abscessus*. Using a laboratory developed whole genome sequencing (WGS) test, the bacterium was determined to be the same strain with the same resistance mechanisms, indicating a relapse. This was deemed a critical element of clinical information, as the isolation of a genetically distinct organism would have indicated a new infection and would have served as evidence that a 12-month regimen was likely sufficient to achieve eradication. The confirmation of a relapse prompted the prolongation of the therapy plan to a goal of 24 months. Reinfection and relapse are great challenges in patients with cystic fibrosis who may acquire new strain of *M. abscessus* from the environment, may harbor multiple subpopulations of bacteria, or may have persistent infections but intermittent bacteria shedding that could not be eradicated. WGS has emerged as a powerful molecular tool to accurately differentiate re-infection from relapse thus solving this conundrum.

## Highlights

▪ Recurrent *M. abscessus* infections are great challenges in cystic fibrosis patients.
▪ WGS enables high-resolution phylogenetic comparison of *M. abscessus* differentiating re-infection from relapse.
▪ Differentiating re-infection from relapse can be useful in managing recurrences of *M. abscessus* infections.

## Introduction

*Mycobacterium abscessus* has emerged as a major threat in patients with cystic fibrosis (CF) and is associated with more advanced lung disease and rapid decline in lung function [1]. In the management of *M. abscessus* infection, sustained sputum culture conversion without relapse is associated with good outcome [2], while reinfection can lead to treatment prolongation and is frequently associated with multiple genotypes [3]. We present a case of a pediatric cystic fibrosis patient with chronic *M. abscessus* infection, where whole genome sequencing (WGS) aided in accurate subspecies identification, phylogenetic relatedness determination and genotypic prediction for drug susceptibility, thereby guiding the clinical management.

## Case report

**Fig. 1:**
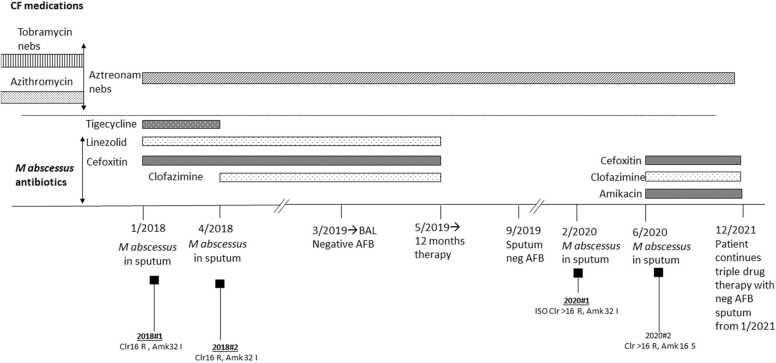
Clinical history, microbiological findings, and antimicrobial treatment timeline.

A 7-year-old female with history of CF and past medical history of failure to thrive, meconium ileus, volvulus, presented with exacerbation of endobronchitis. The pulmonary exam was significant with the presence of diffuse rales and rhonchi, and chest X-ray (CXR) showed new abnormal findings. The patient was receiving azithromycin prophylaxis since 3 years of age as part of the CF medication regimen. Acid-fast bacilli (AFB) culture of sputum grew *M. abscessus* in January and April 2018, and both isolates were resistant to clarithromycin, doxycycline, TMP-SMX, ciprofloxacin and intermediate to cefoxitin and amikacin. Given the extensive drug resistance, declining lung function, and limited treatment options, clofazimine was considered as an investigational drug. Its susceptibility was tested at a reference laboratory, with the minimum inhibitory concentration (MIC) determined to be ≤ 0.5 μg/mL. Treatment was started on January 2018 with tigecycline 25 mg IV q12h, linezolid 10 mg/kg IV q8h and cefoxitin 3500 mg via continuous infusion. After obtaining a single subject emergency IND from the FDA, clofazimine 50 mg orally 3 times a week was started in April 2018 and tigecycline was stopped (Fig. 1). The patient completed 12 months of the clofazimine, linezolid and cefoxitin regimen, and repeat respiratory AFB cultures during therapy and at the end of therapy were all negative. CXR showed gradual resolution of right apical lesions with symptom improvement and pulmonary function test (PFT) improvement. The patient tolerated the antibiotic regimen well, toxicity monitoring test results were obtained weekly throughout the course of treatment did not show any significant abnormalities. The patient did not experience significant skin darkening commonly seen with clofazimine, or significant GI side effects. Her appetite, caloric intake and growth pattern remained within the expected range for her underlying disease. A repeat sputum culture in September 2019 was also negative.

In February 2020, *M. abscessus* grew again in her sputum and her CXR showed new left lower lung focal consolidation. An isolate was sent to a reference laboratory, where clofazimine susceptibility testing was performed, showing the MIC was still ≤0.5 mcg/ml. After renewal of the single subject emergency IND from the FDA, a 3-drug regimen of clofazimine 50 mg orally 3 times a week, cefoxitin 5000 mg via continuous infusion and amikacin 25 mg/kg 3 times a week (coinciding with clofazimine doses) was started in June 2020. Patient again tolerated the antimicrobial regimen; amikacin levels, checked routinely, have remained appropriate, with peaks from mid-40s to mid-50s (μg/mL), and trough always below detection. No abnormalities were observed in her skin color, auditory function, urine color, hematologic parameters liver and kidney function.

A clinical whole genome sequencing (WGS) test routinely performed in the UCLA clinical microbiology laboratory [4] was performed on the *M. abscessus* isolate obtained in 2020 (Isolate 2020#1) and the initial 2 isolates from 2018 (Isolate 2018#1 and 2018#2) for genomic characterization to differentiate reinfection versus relapse. *K-mer* based phylogenetic analysis identified all three isolates as *M. abscessus* subsp*. massiliense*. Single nucleotide polymorphism (SNP) analysis identified only 3 SNPs between Isolate 2020#1 and the 2 genetically identical isolates from 2018 (Isolate 2018#1 and 2018#2), indicating that this patient had a relapse of her previous *M. abscessus* infection, thus not a re-infection. Genotypic resistance prediction analysis showed despite a truncated non-functional *erm(41)* gene, all the 3 isolates possess a A2270T (E. coli numbering) mutation in the 23S rRNA gene *rrl*, which confers to constitutive resistance to clarithromycin, consistent with the phenotypic drug susceptibility results. The availability of genomic results identifying the *M. abscessus* infection as a recurrence rather than a new infection was a critical element of clinical information, as the isolation of a genetically distinct organism would have indicated a new infection and would have served as evidence that a 12-month regimen was likely sufficient to achieve eradication. The confirmation of a relapse prompted the prolongation of the therapy plan to a goal of 24 months. As of the time writing this manuscript, the patient’s CXR in November 2021 showed resolution of focal consolidation and resolved pneumatoceles, coarse interstitial and peribronchial opacities likely due to cystic fibrosis associated changes. The antibiotic regimen has been continued with a goal to complete 24 months if the regimen continues to be well tolerated.

## Discussion

WGS enables high-resolution phylogenetic comparison of the *M. abscessus* isolates in CF patients with chronic infections. In this case, we highlight the value of its capability in differentiating re-infection from relapse by using SNP analysis of the whole genome sequences, which showed only 3 SNP difference between isolates from 2 years apart, indicating that this is a relapse of the same bacteria and there may be a nidus of infection in the lungs which was not cleared in this complex setting. Although there are currently no validated SNP cutoffs for *M. abscessus* to differentiate re-infection from relapse, studies have shown up to 6 SNPs could be seen in persistent infection [5], which is consistent with our findings. None of the 3 SNPs in our case was found to be located in a gene or loci known to be associated with drug resistance; this information helped rule out the possibility of drug resistance that might developed during the 1st round of 12-month treatment course and reinsured the treatment decision for a 24-month regimen.

To our knowledge, this is the first study utilizing WGS for genotyping to differentiate *M. abscessus* reinfection vs. relapse in the clinical setting and in a real-time manner. Another significant non-TB mycobacteria (NTM), *Mycobacterium avium* complex (MAC), also frequently causes chronic pulmonary infection; a previous study used pulsed-field gel electrophoresis (PFGE) to genotype MAC to differentiate reinfection vs. relapse and found that such approach can be helpful in clinical management of MAC recurrent infections [6]. However, WGS represents a superior method than PFGE for genotyping with higher accuracy [7], [8].

This case study demonstrated a proof of concept for the clinical utility of WGS for high-resolution genomic characterization of *M. abscessus* in a chronic infection to help answer critical questions including 1) whether a persistent infection is due to re-infection or relapse; and 2) whether any drug resistance conferring mutation emerges along treatment course. Further studies are needed to fully evaluate the clinical utility of performing WGS for persistent *M. abscessus* infections in the CF patients.
